# Age-related normal structural and functional ventricular values in cardiac function assessed by magnetic resonance

**DOI:** 10.1186/1471-2342-13-6

**Published:** 2013-02-07

**Authors:** Michael Fiechter, Tobias A Fuchs, Catherine Gebhard, Julia Stehli, Bernd Klaeser, Barbara E Stähli, Robert Manka, Costantina Manes, Felix C Tanner, Oliver Gaemperli, Philipp A Kaufmann

**Affiliations:** 1 Department of Radiology, Cardiac Imaging, University Hospital Zurich, Zurich, Switzerland; 2 Department of Cardiology, Cardiovascular Center, University Hospital Zurich, Zurich, Switzerland; 3 Zurich Center for Integrative Human Physiology (ZIHP), University of Zurich, Zurich, Switzerland

**Keywords:** CMR, Age, LV-EF, RV-EF, Normal values

## Abstract

**Background:**

The heart is subject to structural and functional changes with advancing age. However, the magnitude of cardiac age-dependent transformation has not been conclusively elucidated.

**Methods:**

This retrospective cardiac magnetic resonance (CMR) study included 183 subjects with normal structural and functional ventricular values. End systolic volume (ESV), end diastolic volume (EDV), and ejection fraction (EF) were obtained from the left and the right ventricle in breath-hold cine CMR. Patients were classified into four age groups (20–29, 30–49, 50–69, and ≥70 years) and cardiac measurements were compared using Pearson’s rank correlation over the four different groups.

**Results:**

With advanced age a slight but significant decrease in ESV (r=−0.41 for both ventricles, P<0.001) and EDV (r=−0.39 for left ventricle, r=−0.35 for right ventricle, P<0.001) were observed associated with a significant increase in left (r=0.28, P<0.001) and right (r=0.27, P<0.01) ventricular EF reaching a maximal increase in EF of +8.4% (P<0.001) for the left and +6.1% (P<0.01) for the right ventricle in the oldest compared to the youngest patient group. Left ventricular myocardial mass significantly decreased over the four different age groups (P<0.05).

**Conclusions:**

The aging process is associated with significant changes in left and right ventricular EF, ESV and EDV in subjects with no cardiac functional and structural abnormalities. These findings underline the importance of using age adapted values as standard of reference when evaluating CMR studies.

## Background

Cardiac structural and functional ventricular values are important prognostic parameters for predicting patient outcome and are therefore commonly assessed with various methods [1,2] whereby cardiac magnetic resonance (CMR) imaging has emerged as a new gold standard. With an aging Western population, the impact of age on these cardiac parameters is critical for identification of pathology. The ventricular systolic phase represents a synergistic interplay of multiple kinetic components, each of which confers added prognostic information such as short and long-axis shortening and twisting around the ventricular long axis [3]. From the second to seventh decade a substantial decrease in longitudinal shortening (−20%) but an increase in short-axis diameter shortening (+18%) [4] is associated with a prolonged isovolumetric contraction phase [5].

However, the precise impact of aging on the ventricular function has remained controversial. Several studies involving different methods concluded that aging does not have any impact on LV function [6,7], whereas others found a slightly decreased [8-10] LV ejection fraction (LV-EF) in healthy subjects. A recent study assessing cardiac function using echocardiography in a large patient population revealed an increase in LV-EF with a decrease in end-diastolic volume (EDV) and end-systolic volume (ESV) in older compared to younger individuals [11]. A small study using CMR imaging observed an age-dependent increase in LV-EF [12].

However, the impact of age on both LV and in particular right ventricular (RV) function as assessed by CMR has not been investigated in large study populations. The aim of the present study was to compare LV and RV dimensions and EF over consecutive age decades.

## Methods

### Study population

This retrospective study enrolled patients who underwent CMR for assessment of myocardial function including those individuals who had normal function and no cardiac pathology. In addition, the following exclusion criteria were applied: history of any cardiac disease, LV-EF <50% and/or RV-EF <45%, and EDV >235 ml (males) and >174 ml (females) as assessed by CMR [13]. As a result 1016 patients were excluded and 183 patients were included into the final analysis. Study patients were classified into four age groups (20–29, 30–49, 50–69, and ≥70 years). Need to obtain written informed consent was waived by the institutional review board due to the nature of the study with sole clinical data collection.

### Image acquisition and analysis

Supine cardiac magnetic resonance imaging was performed using a 1.5 Tesla magnetic resonance (MR) scanner (Philips Achieva, Best, NL) using electrocardiogram (ECG) triggered breath-hold fast field echo acquisition imaging covering the heart from the valve plane to the apex with a slice thickness of 8 mm with no gap. The field of view was 450 × 400 × 96 mm. Sequence parameters included plane pixel size of 2.1 × 1.8 mm, acquisition time of 25 heart beats, flip angle of 60°, and repetition time/echo time of 3.4/1.7 ms. A cardiac synergy coil was used for signal acquisition and cardiac synchronization was performed with a vector ECG. Standard projections (short axis, two-, three-, and four-chamber view) were obtained for functional and structural assessment. Quantification of left and right ventricular values was performed using a dedicated software and workstation (Extended Work Space, Philips Medical Systems, NL) by two independent CMR observers. In brief, LV and RV endocardial border were delineated in end-systolic and end-diastolic phases in all planes and EDV/ESV were calculated. LV mass was determined from the end-diastolic frames.

### Statistical analysis

Quantitative variables were expressed as mean ± standard deviation (SD) and categorical variables as frequencies or percentages. SPSS 20 (SPSS, Chicago, IL) was used for all statistical analysis. The Pearson’s correlation analysis was used to determine a correlation between the parameters of interest. P-values of less than 0.05 were considered statistically significant.

## Results

This study included 183 subjects with functional and structural assessment of LV parameters. The quantitative RV parameters were available in 111 patients and the LV mass in 147 patients. The mean age of the study population was 52±14.7 years. The mean LV-EF was 64±7.0% while the mean RV-EF was 59±6.6% (Table 1). The most relevant and statistically significant change in LV and RV parameters was observed between the age group 30–49 years and 50–69 years as well as between 50–69 years and ≥70 years.

**Table 1:** Patient baseline characteristics (n=183)


Age (yrs)	52±14.7
Male, n (%)	117 (64)
Mean LV values	
EF (%)	64±7.0
ESV (ml)	50±17.9
EDV (ml)	137±34.2
Mass (g/m^2^)	57±16.7
Mean RV values	
EF (%)	59±6.6
ESV (ml)	52±18
EDV (ml)	126±34.2
Cardiovascular risk factors, n (%)	
Smoking	31 (17)
Hypertension	46 (25)
Diabetes	13 (7)
Dyslipidemia	32 (17)
Positive family history	39 (21)
Medications, n (%)	
Aspirin	50 (27)
Beta-blocker	41 (41)
ACE/angiotensin II inhibitor	43 (23)
Statin	37 (20)
Calcium channel blocker	5 (3)
Nitrate	2 (1)

*LV* left ventricle; *EF* ejection fraction; *ESV* end systolic volume; *EDV* end diastolic volume; *RV* right ventricle; *ACE* Angiotensin converting enzyme.

**Figure 1:**
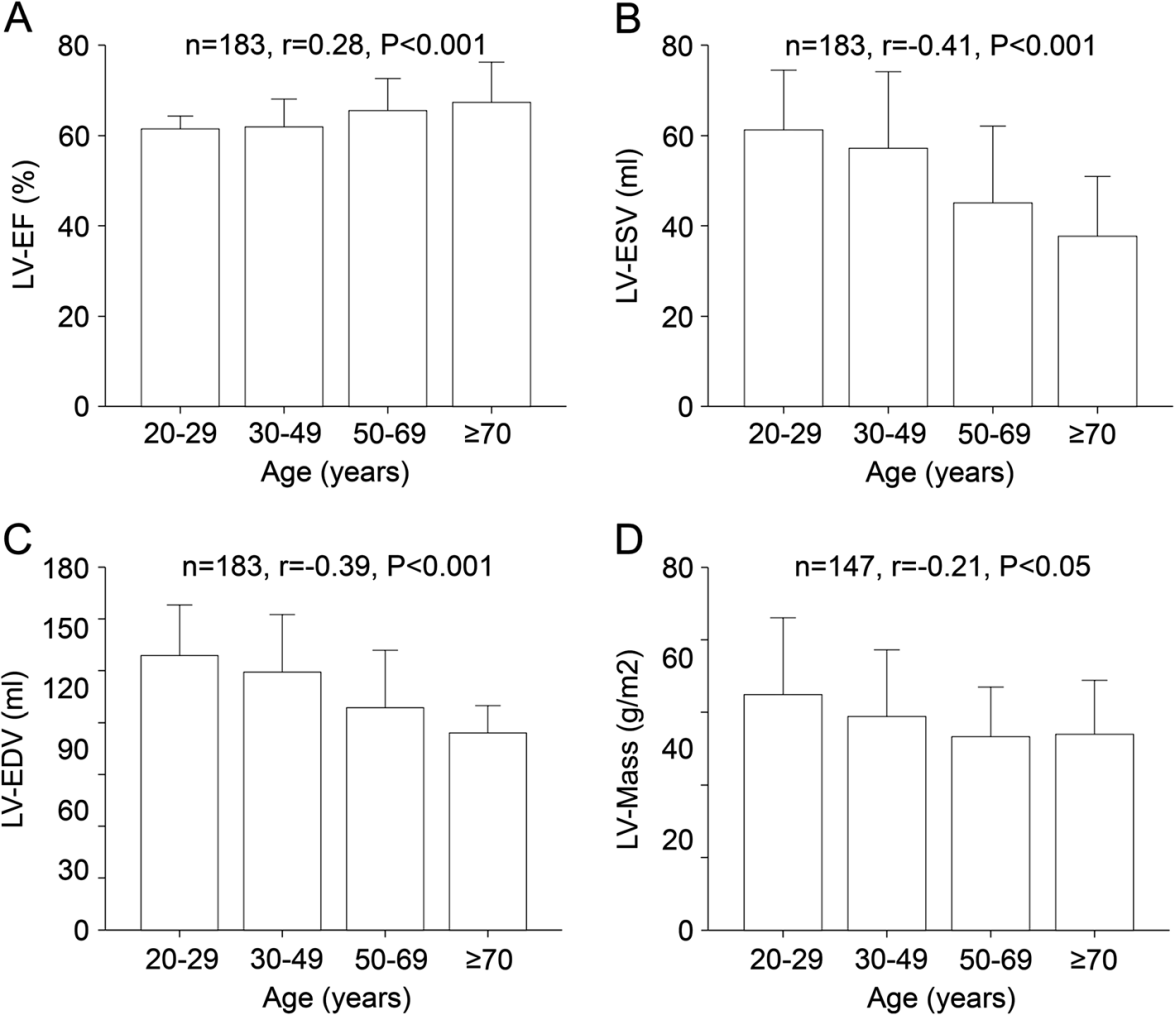
**Age-dependent change of LV values by CMR.** LV-EF (panel **A**) significantly increased over the four age categories whereas LV-ESV (panel **B**), LV-EDV (panel **C**), and LV myocardial mass (panel **D**) significantly decreased with the aging process.

There was a modest but significant correlation of the LV-EF with increasing age (n=183, r=0.28, P<0.001) whereas LV-ESV (n=183, r=−0.41, P<0.001), LV-EDV (n=183, r=−0.39, P<0.001), and LV myocardial mass (n=147, r=−0.21, P<0.05) decreased significantly at advanced age (Figure 1).

**Figure 2:**
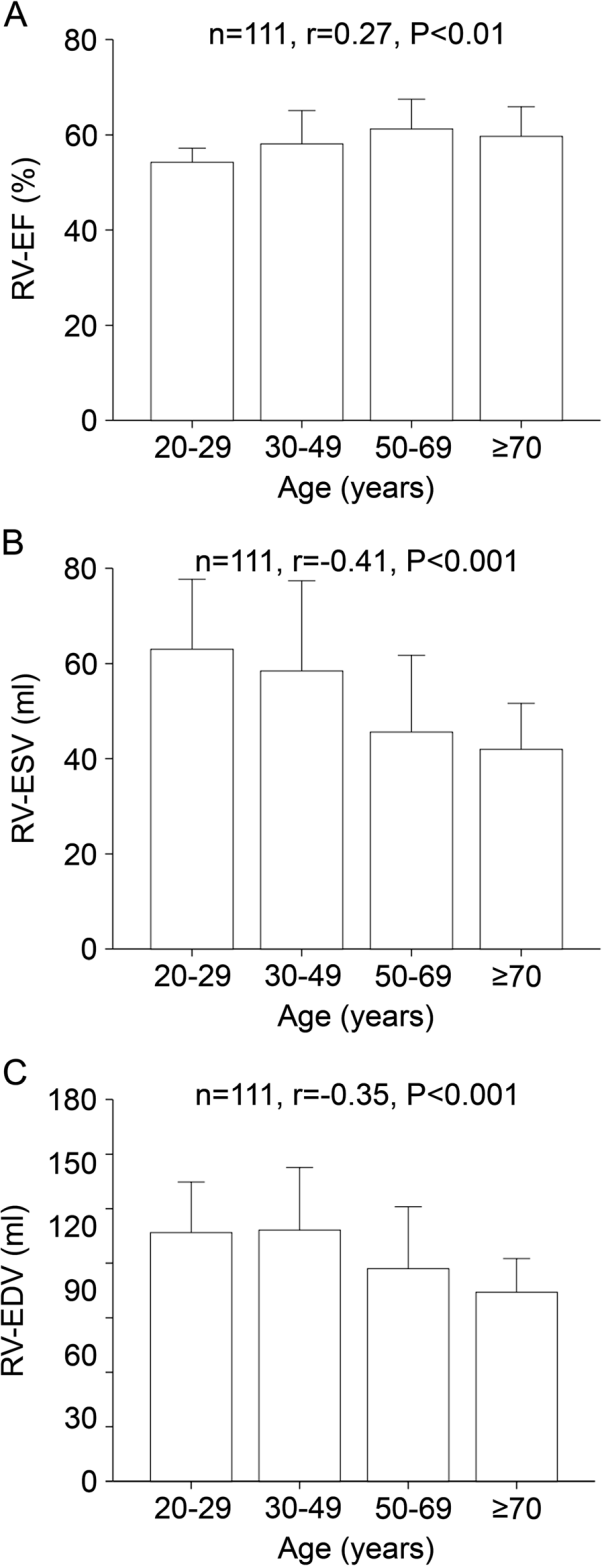
**Age-dependent change of RV values in CMR.** RV-EF (panel **A**) significantly increased over the four age categories whereas RV-ESV (panel **B**), and RV-EDV (panel **C**) significantly decreased with advancing age.

This was paralleled by a significant increase of RV-EF (n=111, r=0.27, P<0.01) whereas RV-ESV (n=111, r=−0.41, P<0.001) and RV-EDV (n=111, r=−0.35, P<0.001) significantly decreased with increasing age (Figure 2).

The results in the different age groups are summarized in Table 2.

**Table 2:** LV and RV measurements in increasing age categories

	**20-29 years**	**30-49 years**	**50-69 years**	**≥70 years**	**P-value**
**LV**	(n = 17)	(n = 61)	(n = 86)	(n = 19)	
EF (%)	60.9±3.2	60.8±5.7	65.2±7.0	69.3±8.7	<0.001
ESV (ml)	63.2±15.2	59.0±17.1	45.1±16.0	35.2±10.4	<0.001
EDV (ml)	161.3±34.6	149.8±33.9	127.6±30.7	114.4±7.6	<0.001
Mass (g/m^2^, n)	62.6±19.9 (16)	56.3±16.2 (52)	50.0±11.2 (63)	52.9±13.9 (16)	<0.05
**RV**	(n = 12)	(n = 43)	(n = 41)	(n = 15)	
EF (%)	54.1±3.1	57.6±6.8	60.9±6.4	60.2±6.3	<0.01
ESV (ml)	62.4±15.2	59.5±18.7	45.5±16.0	41.4±10.4	<0.001
EDV (ml)	135.0±28.5	139.4±34.7	115.3±31.4	103.6±19.4	<0.001

*LV* left ventricle; *EF* ejection fraction; *ESV* end systolic volume; *EDV* end diastolic volume; *RV* right ventricle.

## Discussion

The present CMR study documents that increasing age is associated with a significant decrease in LV and RV volumes but an increase in EF. Furthermore, LV myocardial mass significantly decreased over the four age groups. There is increasing awareness on the fact that changes in cardiac ventricular structure and function attributable to the aging process may play an important role in modulating the cardiovascular response to disease [7]. The knowledge of age-dependent normal structural and functional cardiac values is critical for identification of pathology and prediction of patient outcome. The rapid growth of the elderly population renders definition of normal values in advanced age even more important and may particularly help to reduce expensive and unnecessary follow-up examinations while there is a broad consensus on the decline in diastolic function with age. Controversial findings exist for age-related structural and functional cardiac values ranging from increase over no correlation to a decrease in LV-EF with advancing age [7,8,11,14], mainly obtained from echocardiographic studies. In fact, despite several limitations echocardiography has been the modality applied in most of these reports, reflecting the multi-purpose use of this widely available non-invasive tool. Comparable data as assessed from CMR are limited and particularly scarce for the RV. This is particularly important in view of the fact that in the past decade CMR has emerged as a new gold standard of non-invasive LV and RV assessment which is now increasingly available while the evaluation of mechanistic insights in metabolic and pathophysiological mechanisms remains a privilege of highly sophisticated CMR techniques such as tagging and spectroscopy [15].

Differences in myocyte loss on the one hand and trophic effects on the other hand may eventually shift the tip of the balance towards changes in EF in the aging process. The observed decline in ventricular dimensions seems to favourably counterbalance the decrease in LV muscle mass resulting in an increase in EF. This is supported by the observation that twisting and torsion of the left ventricle are more pronounced in the elderly population [16] and it may help explaining recent observations describing that normal EF can be present despite impaired myocardial contractility [17]. While our results on a decrease in LV volume are in line with the majority of echocardiography and CMR studies, the findings with regard to LV mass seem more controversial. In some studies, LV mass was unchanged during aging [12] while in other studies an increase was found [14,18,19]. Interestingly, there was no association between age and LV mass in the Framingham study detectable by echocardiography [20], while findings were described in a recent CMR analysis of the Framingham study [21] showing a decrease over age. As the latter revealed LV mass as an important predictor of morbidity and mortality [18], a decrease in LV mass could also reflect a survival selection bias. Due to its cross-sectional design our study avoids such bias and provides data from a real live cohort. However, for adequate interpretation it is crucial to review the techniques used, as the results may not necessarily be interchangeable [22]. As mentioned above, most previous studies have assessed LV mass by M-mode echocardiography based on models which take into account LV wall thickness and short-axis dimensions neglecting the real LV shape. In addition, this method is dependent on operator skills. A recent study using latest advancements in echocardiography, i.e. real time 3-dimensional echocardiography, found the lowest values for LV mass and volumes in the eldest patients (seventh decade of life) largely in line with our results. This was further supported by other observations using CMR [23,24].

We acknowledge the following potential study limitations: First, in line with the data mentioned above we present a cross-sectional study of individuals at various ages, whereas patients would need to be followed in time to be more conclusive about age-related changes in cardiac parameters. Second, we did not verify our data with values obtained from another imaging modality and thus are not able to exclude potential modality bias. However, CMR is a well-accepted gold standard itself for such measurements [12]. Third, despite the fact that this is the largest study analysing structural and functional cardiac values in CMR a potential bias towards supernormals as well as a survival selection bias cannot be excluded with final certainty. Furthermore, the limited sample size within subgroups of age does not allow gender-specific analysis. Fourth, we did not standardize loading conditions which may potentially have affected our measurements. However, in healthy individuals the impact of variations in loading conditions appears limited within a broad range of physiologic conditions. In addition, measurements without active interaction by the observer may prevent distortion of the findings closely reflecting real life conditions. Finally, we did not correct the chamber volumes for body size although an association between an increase in body surface area and an increase in chamber dimensions has been reported. However, the aim of the present study was to evaluate the structural and functional changes of the ventricles. As the aging process is per se associated with a decrease in body size normalization of the volumes would have at least in part masked the changes in ventricular volumes observed with aging. This would render meaningful comparison with changes in EF difficult, as for the calculation of EF the normalization cancels out resulting in an uncorrected value [25,26].

## Conclusions

In conclusion, the aging process has significant impact on ventricular EF, ESV and EDV in healthy CMR patients with normal functional and structural cardiac values. These findings underline the importance of using age adapted values as standard of reference in CMR studies.

## Competing interests

The authors have no competing interests to declare.

## Authors’ contribution

MF, TAF and PAK were substantially involved in conceptual design of the study, statistical analysis of the data and drafting of the manuscript. CG, JS, BK, and BS were involved in critical review of data integrity, and gave their intellectual input concerning manuscript drafting. RM, CM, TAF and MF were substantially involved in acquisition of imaging data in daily clinical routine. Further, RM and CM gave substantial technical input regarding cardiac magnetic resonance imaging. FT, OG, and PAK critically reviewed the manuscript and gave important intellectual input. All authors have critically reviewed and approved the final version of the manuscript.

## Pre-publication history

The pre-publication history for this paper can be accessed here:

http://www.biomedcentral.com/1471-2342/13/6/prepub
